# Extensive Surgical Emphysema Following Stab Injury to the Neck

**DOI:** 10.7759/cureus.20126

**Published:** 2021-12-03

**Authors:** Jouhar J Kolleri, Akram Al-warqi, Rowaa I Mohamed, Ali Khaliq, Salman Mirza

**Affiliations:** 1 Clinical Imaging, Hamad Medical Corporation, Doha, QAT; 2 Anesthesia, Hamad Medical Corporation, Doha, QAT

**Keywords:** pneumoperitoneum, penumomediastinum, pneumopericardium, pneumothorax, tracheal injury, penetrating injury, stab injury, extensive surgical emphysema

## Abstract

Extensive surgical emphysema can lead to a life-threatening condition causing hemodynamic instability and significant physician challenges in its management. Here we describe an uncommon case of extensive subcutaneous emphysema caused by stabbing neck, which led to complications such as pneumothorax, pneumopericardium, pneumomediastinum as well as pneumoperitoneum. The role of radiological imaging is crucial in managing this relatively uncommon presentation. This article highlights clinical presentation, radiological findings, and various management options.

## Introduction

The presence of air in the subcutaneous area is defined as surgical emphysema [1]. Tracheal injury may result in extensive subcutaneous surgical emphysema, and potentially critical conditions like tension pneumothorax, mediastinal emphysema, stridor, and respiratory distress in patients. Extensive surgical emphysema following penetrating injury to the trachea after a suicide attempt is a case that is rarely reported in the literature to the best of our knowledge. Management of tracheal injury is difficult, and emergency physicians, anesthesiologists, and surgeons all play a role in the patient's care. We present the case of a 24-year-old lady who had a tracheal rupture after attempting suicide by stabbing her throat.

## Case presentation

A 24-year-old lady was brought to the emergency department after an alleged suicide attempt by stabbing her neck with a knife. She was intubated on the scene by the emergency medical services team and transferred to the Trauma resuscitation unit. On examination, her vitals were as follows: temperature: 36.3 degrees Celsius, blood pressure: 141/65 mm Hg, heart rate: 90/minute, respiratory rate: 20/minute, oxygen saturation: 99% on the ventilator with fraction of inspired oxygen (FiO_2_) 100%. Upon general examination, she was conscious and oriented, pupils equal and reactive, and moving all limbs. On local examination, a knife was seen penetrating the right side of neck. She had extensive subcutaneous edema in the chest, neck, and face. There was a 3 cm transverse laceration to the neck. No vascular injury was seen. Respiratory examination revealed bilateral decreased breath sounds.

**Figure 1:**
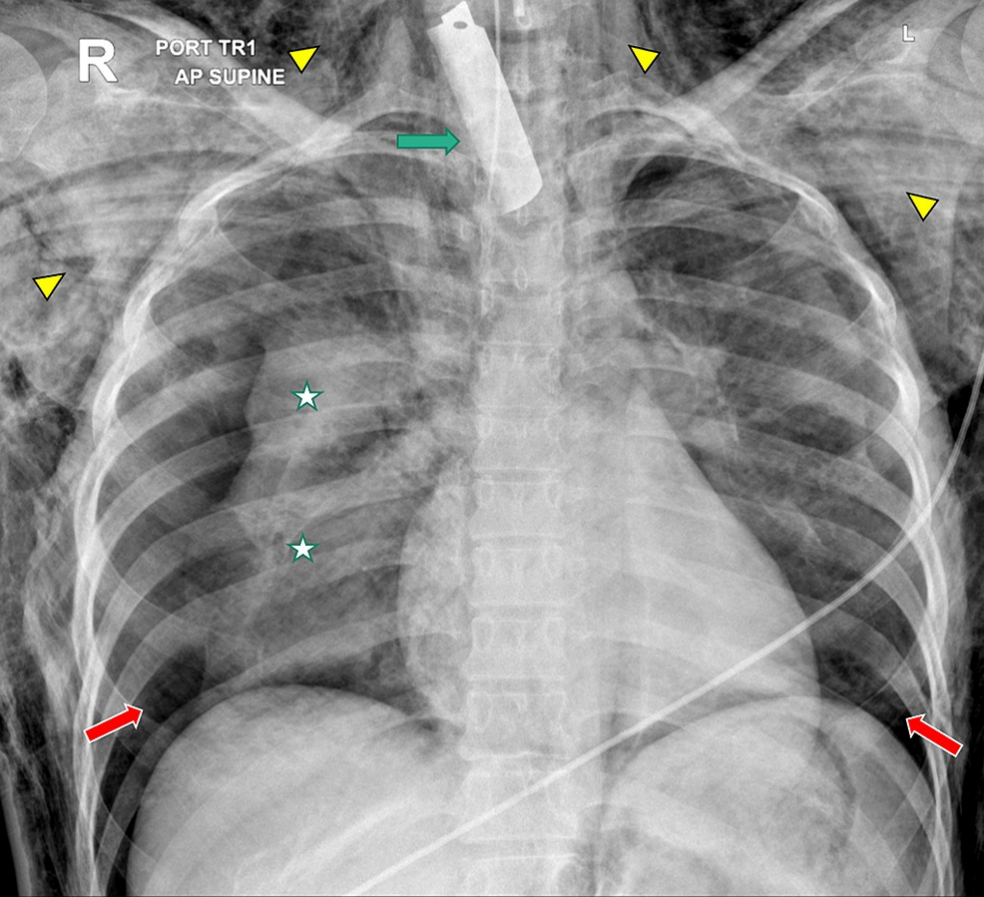
Chest X-ray showing a larger rectangular radiopacity seen projecting over the root of the neck extending to the thoracic inlet in the midline concerning for a foreign body (green arrow), bilateral pneumothorax right larger than the left (red arrows ) with atelectasis of the right lung (star), and extensive surgical emphysema in the chest wall (yellow arrowhead).

Chest X-ray showed bilateral pneumothorax, more on the right side with atelectasis of right lung (Figure 1). Bilateral chest tubes were inserted. The patient was shifted to the operating room immediately for neck exploration. There was a 3 cm transverse laceration to the trachea which was repaired. Postoperative diagnosis is tracheal laceration following stab injury to the neck. She was started on intravenous ceftriaxone for 5 days.

**Figure 2:**
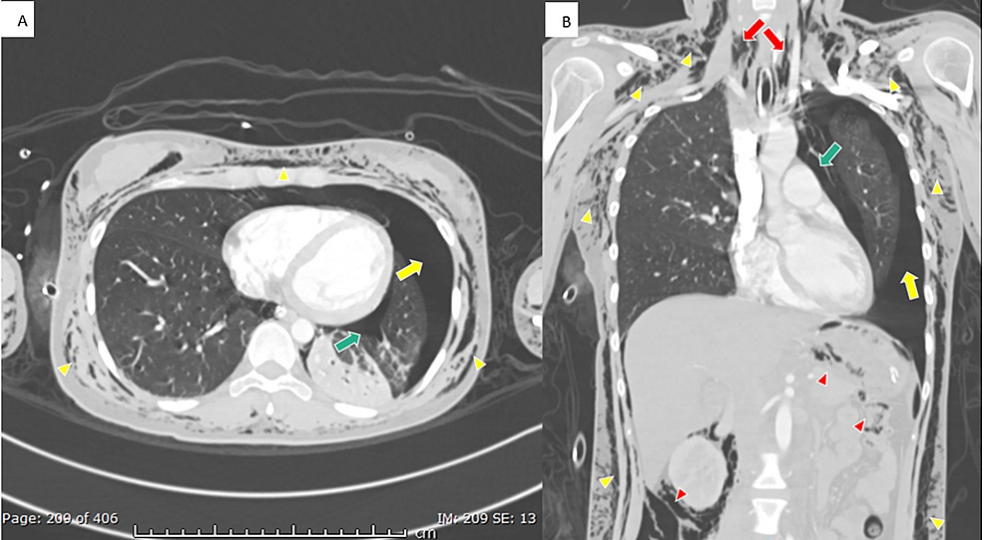
CT chest A) axial and B) coronal CT chest in the lung window showing diffuse subcutaneous surgical emphysema (yellow arrowhead), large left pneumothorax (yellow arrow), moderate amount of pneumopericardium (green arrow), pneumomediastinum (red arrow), and large amount of intra-abdominal air (red arrowhead). CT, computed tomography.

Computed tomography of head, neck, chest, and abdomen was done on the same day, which showed diffuse subcutaneous emphysema in the neck, head region reaching till the vertex, and along the chest and abdominal wall extending to the visualized proximal lower limbs. Large left pneumothorax with minimal right pneumothorax was seen. A moderate amount of pneumopericardium, pneumomediastinum, and pneumoperitoneum was also noted (Figure 2).

**Figure 3:**
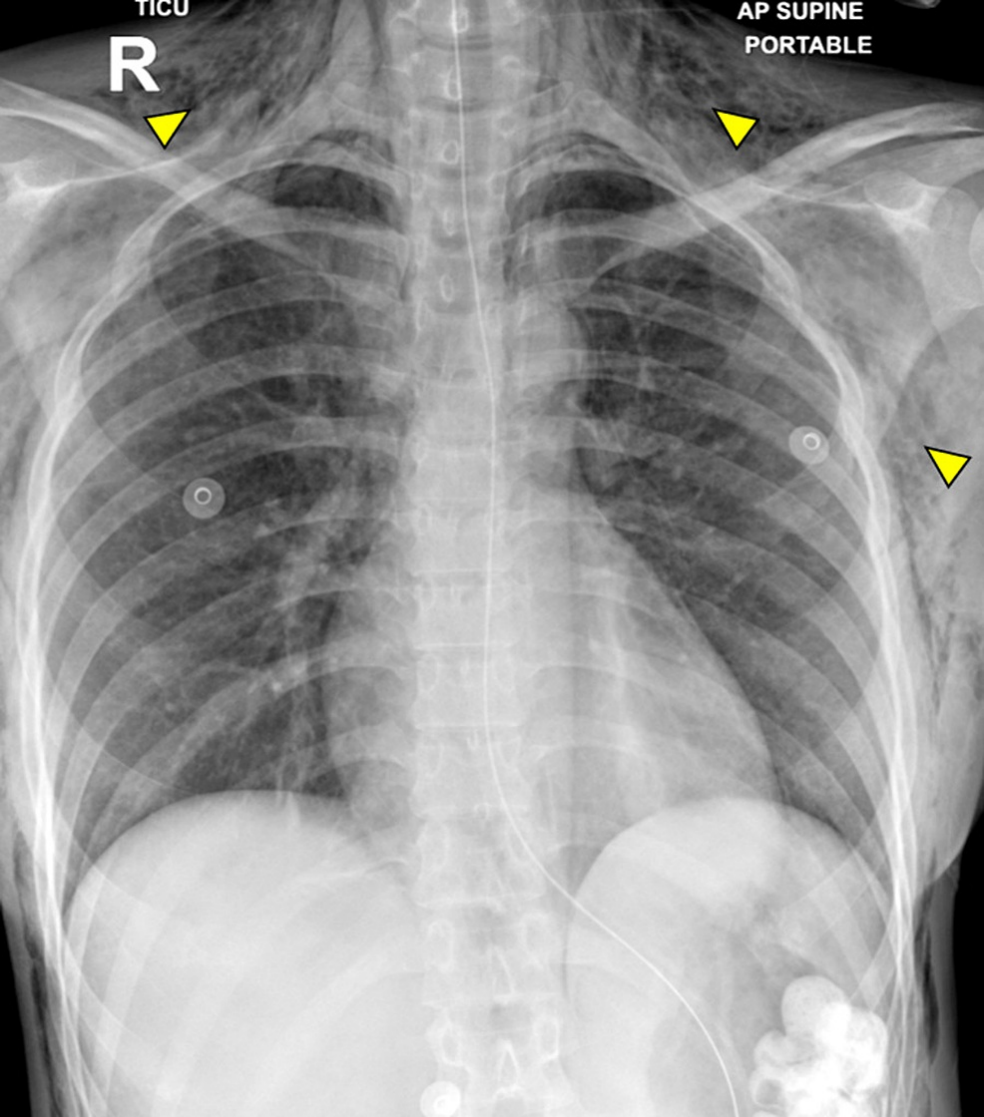
Chest X-ray showing significant resolution of the bilateral pneumothorax with residual subcutaneous emphysema (yellow arrow).

During her hospital stay, the subcutaneous emphysema was resolving, and a repeated chest X-ray showed improvement, and a chest drainage tube was removed on day 3 (Figure 3). She was extubated successfully on day 5 of admission. Her vitals were within normal limits and tolerating an oral diet. Her medical recovery was satisfactory, and she was transferred to a psychiatry hospital on day 8 for further assessment.

## Discussion

Any injury that occurs between the cricoid cartilage and the carina is referred to as a tracheal rupture. It can be caused by iatrogenic causes, blunt chest trauma, piercing wounds, avulsion, or explosive injuries [2]. Sudden deceleration following a high-speed car accident is the most common cause of post-traumatic damage [2,3]. However, a suicidal attempt is a rare cause of tracheal rupture in the emergency department since it is not a common entity.

Swelling and crepitus over the affected area are typical signs of spontaneous subcutaneous emphysema. Other signs and symptoms may include a scratchy throat, stiff neck, trouble swallowing, breathing, and abdominal distension. As a result, it usually manifests as minor symptoms and poses no health risk on its own. The condition will be serious, distressing, and even fatal if it affects the deeper tissues of the thoracic outlet, chest, or abdominal wall. High airway pressure, severe respiratory acidosis, respiratory failure, and tension pneumothorax can result if full lung re-expansion is restricted [4].The clinical feature depends on the extent and degree of the emphysema and the patient's overall health.

Imaging helps to confirm the diagnosis in questionable situations, exclude local related problems, determine the extent, and track the progression of the emphysema. Intermittent regions of radiolucency on a radiograph, often representing a fluffy appearance on the external borders of the thoracic and abdominal walls, can be seen. In extensive cases, air is seen outlining major organs. Chest X-rays are most usually used to diagnose subcutaneous emphysema, pneumothorax, and pneumomediastinum, with the exception of tiny gas collections that can only be detected via a CT scan of the chest. A whole-body CT helps us to assess the extent of displaced gas to various body areas.

There is debate regarding the best way to treat tracheal lesions. The size and location of the lesion are important considerations when deciding on the best treatment method [3]. For tears under 2 cm, some facilities recommend a more conservative approach to treatment [5]. Breathing oxygen may speed up the body's absorption of subcutaneous air. Small wounds or "blow holes" can be made in the skin to release the air [6]. Catheters can be inserted into the subcutaneous tissue to release air in severe cases of surgical emphysema [4].

For major lacerations, conservative treatment is out of the question due to the increased risk of infection and inflammation [7]. The failure of conservative treatment (requiring late repair) is usually due to the persistent passage of air and tracheobronchial secretions through the laceration into the mediastinum leading to mediastinal emphysema, mediastinitis, sepsis, and abscess formation, ventilatory difficulty, pseudo diverticular formation at the laceration site, and formation of fistulae. Acute mediastinitis is a serious infection that often leads to death due to its quick clinical course. Symptoms such as high fever, and discomfort in the chest, back, or upper abdomen can be suggestive of underlying mediastinitis. It is important to fix the wound as soon as possible, supplemented by using a wide range of antibiotics and supportive care [8].

Some studies recommend an elective tracheotomy to prevent airway pressure build-up, thereby avoiding major airway injuries while maintaining spontaneous respiration because increased airway pressure may push the air and secretion to pass through the tear into the mediastinum. Coughing, Valsalva maneuver, sneezing, laughing, and speaking loudly all raise tracheal pressure, resulting in a continuous fissure margin splitting that impedes the healing process and sets the stage for fistula formation. Due to inadequate lung function, positive pressure ventilation may be the only option in some cases. Reduced peak airway pressure, lower tidal volume, and a higher respiratory rate can all be used in this situation to lessen the likelihood of an airway leak reopening [8].

Management of pneumopericardium usually depends on the physiological parameters of the patient. If the patient is stable, it can be managed conservatively. On the other hand, if unstable, decompression can be done. For pneumothorax, the management involves passing a chest tube to get rid of the trapped air [9]. Similarly, minimal pneumomediastinum and pneumoperitoneum can also be treated conservatively. Free air in body cavities has several treatment options depending on where it is located and how severe the condition is. Free air was found in the pleural cavity, mediastinum, peritoneum, pericardium, and subcutaneous tissue of our patient. Emphysema in these regions has been treated by oxygen at high concentrations. It is reabsorbed into capillaries by diffusion along a partial pressure gradient created by the sum of the partial pressures of water, carbon dioxide, nitrogen, and oxygen in pneumothorax, subcutaneous, and mediastinal emphysema. The gradient for gas absorption increases four to six times when breathing 100% oxygen because nitrogen is flushed out of the circulation [10,11]. FiO_2_ 100% was given to our patient for 5 days. The subcutaneous emphysema, pneumopericardium, pneumoperitoneum, and pneumomediastinum improved over time, as evidenced by repeated radiographs. The chest drainage tube was removed after three days, extubated on day 5, and she got symptomatically better and was discharged to psychiatry unit with trauma clinic follow-up.

## Conclusions

The rarity of the etiology in our case makes it an uncommon presentation. A thorough physical examination and review of images are used to make the diagnosis in the appropriate clinical setting. Imaging is extremely beneficial in accurately diagnosing pneumothorax, pneumomediastinum, and soft tissue emphysema because it enables us to determine the precise extent of gas infiltrating different bodily parts. An early diagnosis and apt management reduce the morbidity and mortality considerably.
